# Prior information use and response caution in perceptual decision-making: No evidence for a relationship with autistic-like traits

**DOI:** 10.1177/17470218211019939

**Published:** 2021-05-25

**Authors:** Chris Retzler, Udo Boehm, Jing Cai, Aimee Cochrane, Catherine Manning

**Affiliations:** 1 Department of Psychology, University of Huddersfield, Huddersfield, UK; 2 Department of Psychology, University of Amsterdam, Amsterdam, The Netherlands; 3 Department of Experimental Psychology, University of Oxford, Oxford, UK; 4 School of Psychology and Clinical Language Sciences, University of Reading, Reading, UK

**Keywords:** Autism spectrum, perceptual decisions, motion processing, diffusion model

## Abstract

Interpreting the world around us requires integrating incoming sensory signals with prior information. Autistic individuals have been proposed to rely less on prior information and make more cautious responses than non-autistic individuals. Here, we investigated whether these purported features of autistic perception vary as a function of autistic-like traits in the general population. We used a diffusion model framework, whereby decisions are modelled as noisy evidence accumulation processes towards one of two bounds. Within this framework, prior information can bias the starting point of the evidence accumulation process. Our pre-registered hypotheses were that higher autistic-like traits would relate to reduced starting point bias caused by prior information and increased response caution (wider boundary separation). 222 participants discriminated the direction of coherent motion stimuli as quickly and accurately as possible. Stimuli were preceded with a neutral cue (square) or a directional cue (arrow). 80% of the directional cues validly predicted the upcoming motion direction. We modelled accuracy and response time data using a hierarchical Bayesian model in which starting point varied with cue condition. We found no evidence for our hypotheses, with starting point bias and response caution seemingly unrelated to Adult Autism Spectrum Quotient (AQ) scores. Alongside future research applying this paradigm to autistic individuals, our findings will help refine theories regarding the role of prior information and altered decision-making strategies in autistic perception. Our study also has implications for models of bias in perceptual decision-making, as the most plausible model was one that incorporated bias in both decision-making and sensory processing.

## Introduction

Processing visual information and integrating it with prior experience to guide decisions is key to successfully interacting with the dynamic world around us. These processes may function differently in autism, a neurodevelopmental condition characterised by social communication difficulties and repetitive and restricted patterns of behaviour and interests (see Pellicano & Burr, 2012; Robertson & Baron-Cohen, 2017, for reviews). One aspect of visual processing that has received much focus in autism research is motion perception, with some studies reporting reduced motion sensitivity in autism (Manning et al., 2013; Milne et al., 2002; Pellicano et al., 2005), while other studies show improved sensitivity (Chen et al., 2012; Foss-Feig et al., 2013; Manning et al., 2015) or similar performance in autistic individuals relative to neurotypical individuals (Del Viva et al., 2006; Jones et al., 2011). This disparate literature may be due to differences in participant samples and the type of experimental task or stimuli used (see Kaiser & Shiffrar, 2009; Simmons et al., 2009, for reviews). Yet, despite these differences across studies, evidence has emerged for a small reduction in sensitivity in autistic individuals in motion coherence and biological motion tasks which require perceiving the overall motion of a cloud of dots or a point-light figure, respectively (Van der Hallen et al., 2019). These tasks have in common that they require integrating motion signals over space (“global” motion sensitivity).

It is possible that differences between autistic and non-autistic individuals in perceptual tasks will become more pronounced when introducing prior information which can bias responses. Using a Bayesian framework, Pellicano and Burr (2012) suggested that autistic individuals rely less on prior information than neurotypical individuals, and instead rely more on incoming sensory signals. Although this might lead to autistic individuals perceiving the world more veridically in some situations, performance may be adversely affected in others. Developments of this theory have been rooted in the predictive coding framework (see Clark, 2013, for review), where priors and sensory signals are reframed as predictions and prediction errors; again these theories suggest that the balance between prior information and sensory signals is atypical in autism (Friston et al., 2013; Lawson et al., 2014; Van de Cruys et al., 2018).

The current investigation aimed to better understand individual differences in motion processing and decision-making. Specifically, we investigated whether the use of prior information in a motion processing task varies as a function of autistic-like traits in the neurotypical population. This research can inform and guide studies of individuals with a clinical diagnosis of autism because autistic-like traits have been proposed to vary continuously across the population, with autistic individuals lying at one end of this continuum (Baron-Cohen et al., 2001; Constantino & Todd, 2003). Indeed, Grinter et al. (2009) reported that individuals with high levels of autistic-like traits have increased motion coherence thresholds, like diagnosed autistic individuals, suggesting that the results obtained in this study may be relevant for understanding perceptual performance in autism (but see Burghoorn et al., 2020). In addition, some studies have shown relationships between autistic-like traits and prior information use (Aru et al., 2018; Karvelis et al., 2018; Powell et al., 2016; Skewes et al., 2015), suggesting that weaker priors may extend to individuals with high levels of autistic-like traits (but see also Andermane et al., 2020; Tulver et al., 2019).

This study assesses the influence of prior knowledge on motion coherence discrimination using the diffusion model framework (Ratcliff & Rouder, 1998), in which decisions are modelled as noisy evidence accumulation processes towards one of the two decision bounds. The diffusion model provides greater insight regarding the cognitive processes underlying task performance than traditional accuracy and response time (RT) measures, and can provide alternative explanations of the behavioural results. For example, in our own work, the diffusion model has allowed us to conclude that inattentive children’s reduced task accuracy was due to less efficient information processing rather than a speed-accuracy trade-off (Retzler et al., 2020), and that age-related differences in children’s motion sensitivity are driven by changes in both speed-accuracy trade-offs and efficiency of information processing (Manning et al., 2021).

**Figure 1.**
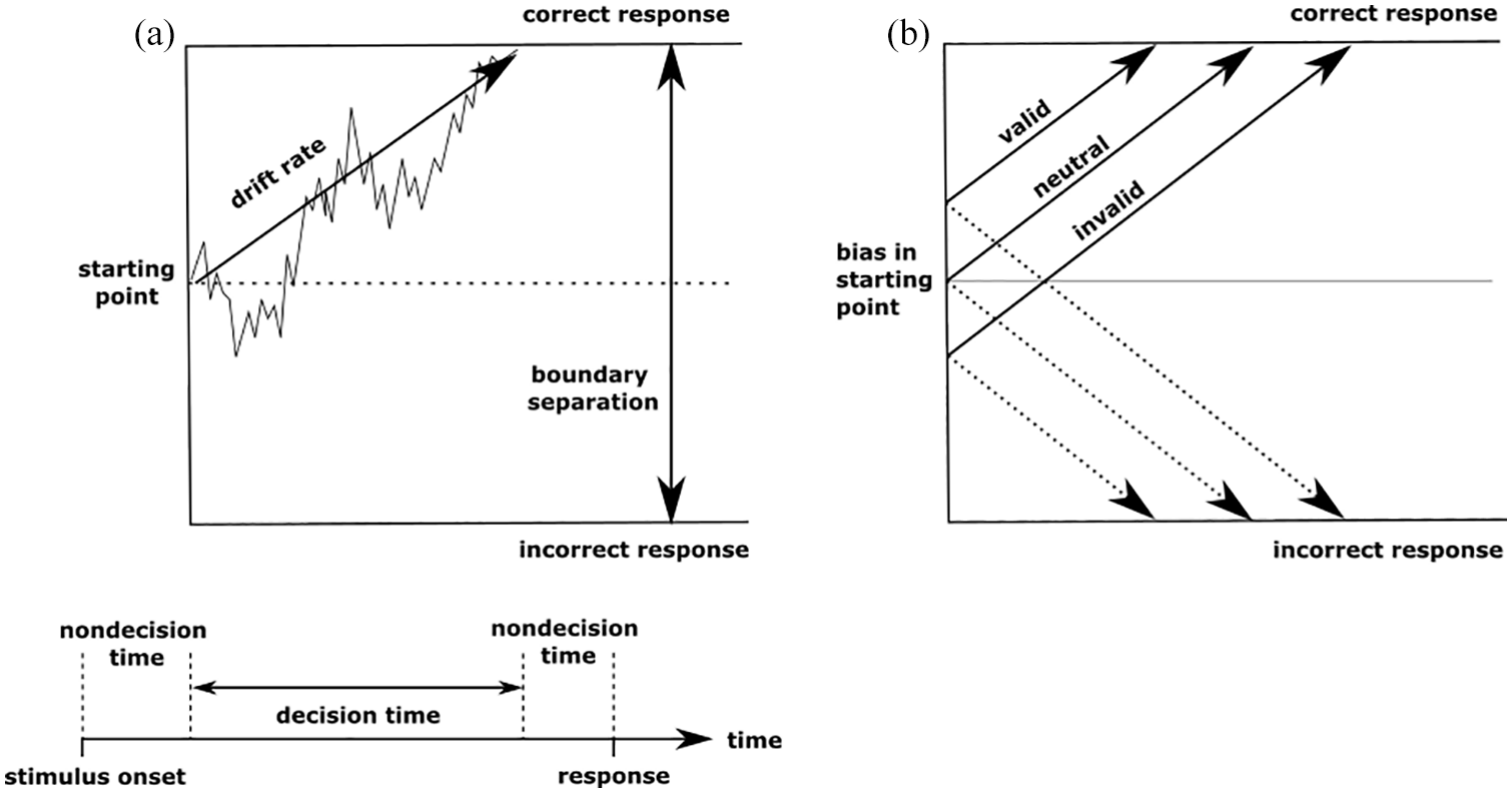
Representation of the diffusion model showing the noisy accumulation of information from the starting point towards a decision boundary (a) and the hypothesised effects of providing valid, neutral, and invalid cues on the starting point (b).

The main parameters of the diffusion model are drift rate, boundary separation, starting point, and non-decision time. The drift rate parameter reflects the efficiency of information processing, which corresponds to the rate of information accumulation over time towards one of two decision boundaries (see Figure 1). Drift rates will be lower during hard decisions, such as when two stimuli are difficult to discriminate, and higher during easier discriminations. The separation of decision boundaries reflects speed-accuracy trade-off, or response caution. Wider boundaries reflect a tendency to require more evidence before reaching a decision, whereas a less cautious approach would be reflected in reduced boundary separation. The position of the starting point in relation to the boundaries determines whether there is a bias towards one response over another. If the starting point is closer to one boundary then there is a greater likelihood of reaching that boundary on a given trial and thus a bias towards that response. If the starting point is equidistant from each boundary then there is no bias towards one response over the other. Finally, non-decision time reflects processes outside of the decision-making process including encoding and response preparation. More complex models with parameters for trial by trial variability in drift rate, non-decision time, and starting point can be constructed but fitting these models requires more data than is typically acquired from behavioural tasks and the parameters of the simplified model used here are the most cognitively interesting (Lerche & Voss, 2016).

The two main ways in which prior information can influence decision-making within the diffusion model framework, are through a change in drift rate (Diederich & Busemeyer, 2006; Ratcliff, 1985), or in a biased starting point (Bogacz et al., 2006; Diederich & Busemeyer, 2006; Link & Heath, 1975; Voss et al., 2004). In cases where the decision-maker is aware of the biasing information prior to the onset of evidence accumulation then we would expect the bias to be reflected in the starting point parameter of the diffusion model (van Ravenzwaaij et al., 2012). For example, Mulder et al. (2012) found that presenting probabilistic directional cues immediately before random dot motion stimuli biased the starting point of the decision-making process in typical adults towards the cued direction (see also Forstmann et al., 2010). Similarly, Rao et al. (2012) trained macaques on a task in which arrow cues indicated the prior probability of stimulus motion. They used activity in the lateral intraparietal area (LIP) to investigate the effects of bias on decision-making, and found that the bias cue changed the initial activity of the LIP neurons (analogous to the starting point) but did not change the scale of sensory evidence (drift rate).

Here, we used a task similar to that of Mulder et al. (2012) to investigate whether the extent of starting point bias following probabilistic cues varied as a function of autistic-like traits in a neurotypical population. We also tested a second hypothesis based on previous research using the diffusion model which has shown increased response caution (i.e., wider boundary separation) in autistic individuals compared with neurotypical individuals (Pirrone et al., 2017; Pirrone et al., 2020). We investigated whether this tendency extends to members of the general population with high levels of autistic traits in our motion task. Interestingly, one previous study that investigated the relationship between autistic traits and diffusion model parameters in a neurotypical population reported no correlation between autistic-like traits and response caution in three tasks, including a motion coherence task (Pirrone et al., 2018). However, this study had a small sample size (*n* = 39) which may have been underpowered to detect an effect.

In this study, we investigated the relationship between autistic-like traits and performance on a random-dot motion paradigm in which prior probability information indicated the likely direction of motion. The accuracy and RT data from this task were then modelled using the diffusion model and the resulting parameters related to autistic-like trait scores using a plausible values approach (Ly et al., 2017, 2018). Our pre-registered hypotheses (https://osf.io/jqpxy/) were that individuals with higher levels of autistic-like traits would show (1) a lesser extent of starting point bias when presented with prior probability information, and (2) wider response boundaries, than individuals with lower levels of autistic-like traits (i.e., a negative and positive correlation, respectively). The difficulty level (stimulus strength) was tailored to each individual’s sensitivity so that we did not expect estimates of drift rate to vary systematically with levels of autistic-like traits.

## Methods

### Participants

A total of 241 participants were recruited primarily from the student populations at the Universities of Huddersfield and Oxford, of which 17 were excluded as they self-reported a diagnosis of a neurological, developmental, or psychiatric disorder, and two were excluded due to equipment malfunction. The remaining 222 participants were aged between 17 and 56 years (*M* = 20.86 years, *SD* = 5.02 years), had self-reported normal or corrected-to-normal vision and were fluent English speakers. Sex data were only recorded for 196 participants of whom 148 were female. The data were collected in undergraduate lab sessions at the University of Huddersfield (*n* = 152) and as part of undergraduate student projects at the University of Oxford (*n* = 70). Total scores on the Adult Autism Spectrum Quotient (AQ; Baron-Cohen et al., 2001) were between 4 and 40 (*M* = 17, *SD* = 6.77). Item-level AQ scores were not recorded for data collected at the University of Huddersfield. Of those who self-reported handedness, 210 were right-handed and 8 were left-handed. Six participants did not complete all five blocks of the task due to time restrictions but were retained in the data set. The study was approved by the ethical review boards at the respective institutions (University of Oxford Medical Sciences Inter-Divisional Research Ethics Committee [R64394/RE002; R64395/RE001]; University of Huddersfield, School of Human and Health Sciences Research Ethics Panel SREP/2017/104) and written informed consent was obtained from each subject.

### Stimuli

**Figure 2.**
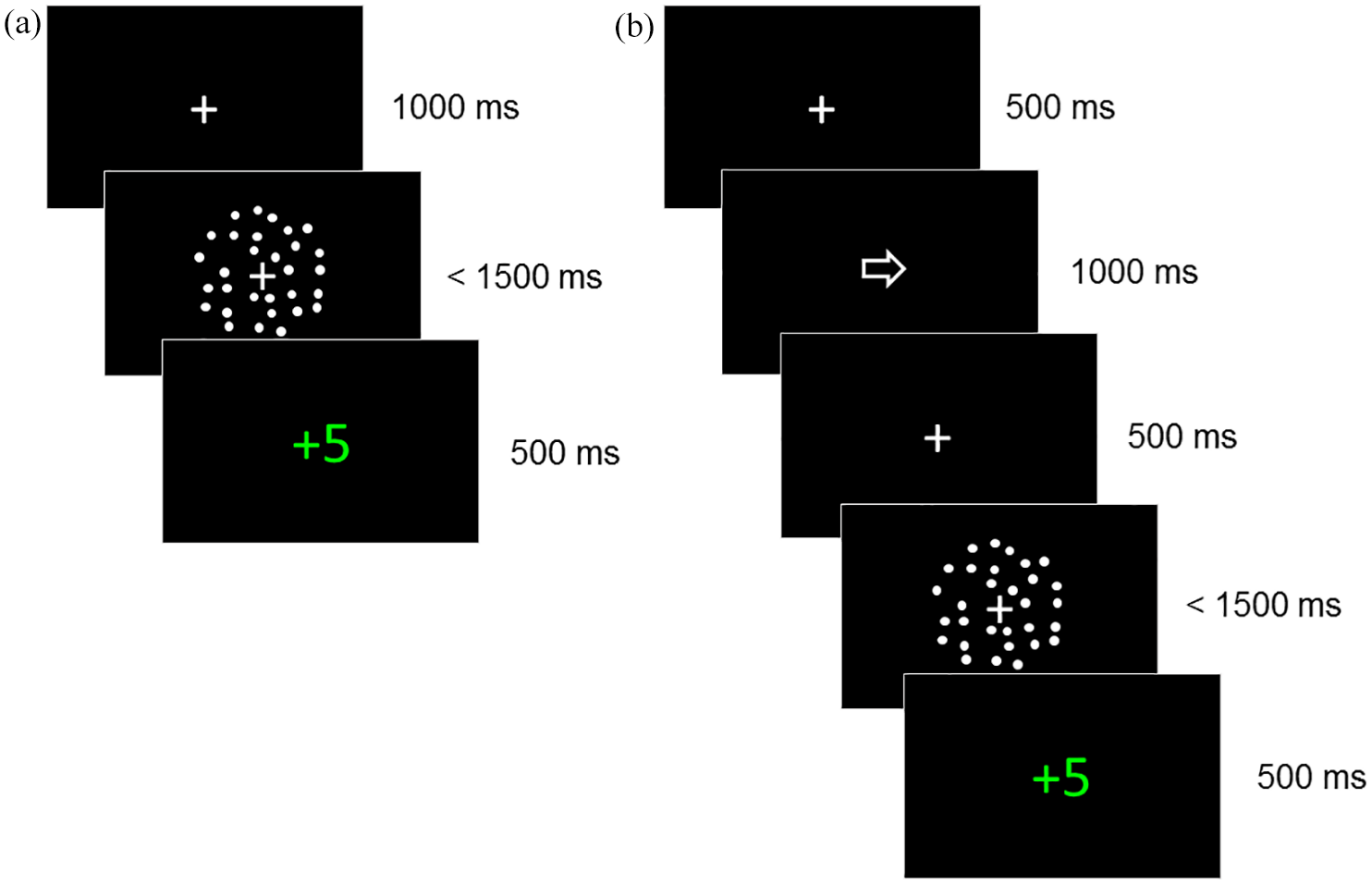
Representation of trial structure in initial staircase phase (a) and main experimental phase (b). (a) Representation of a trial in the initial staircase phase with feedback for a correct response made between 100 and 1,500 ms following the motion stimulus. (b) Representation of a trial in the experimental phase where a directional cue (arrow) was presented before the motion stimulus with feedback for a correct response made between 100 and 1,500 ms.

The task was presented on calibrated monitors with a frame rate of 60 Hz using PsychoPy software (Peirce, 2009; Peirce et al., 2019). Stimuli were white dots (diameter ~.12°) moving on a black background within a circular aperture (diameter 10°), with a speed of 5°/s. An average of 100 dots were presented per frame and each dot “lived” for six frames, after which it was terminated. With each new frame, the dot either moved positions randomly or in line with the motion direction. Dots moving in line with the motion direction were signal dots and those moving randomly were noise dots. The noise dots followed a random but constant direction. A fixation cross (width 0.8°) was present before and during stimulus presentation (see Figure 2).

### Procedure

The task was based on that used by Mulder et al. (2012), and involved participants deciding the coherent motion direction of a random dot pattern. Participants sat 50 cm from the monitor and were instructed to judge whether most of the dots were going to the left or the right, and to indicate their response using the left and right arrow keys on the keyboard. The experiment began with a staircase phase to estimate the coherence level at which the participant responded with an accuracy of 80% correct. This staircase procedure was designed to titrate the difficulty level to each participant’s sensitivity, ensuring that each participant made errors to obtain stable model fits (see also Mulder et al., 2012). In each trial, a fixation dot was presented for 1,000 ms before the motion stimulus, which was presented until the participant responded or until 1,500 ms elapsed (see Figure 2a). Trial-by-trial feedback was given following the participant’s response. If participants responded within 100 and 1,500 ms, they were given 5 points (shown in green text) for a correct response or 0 points (shown in red text) for an incorrect response. Otherwise, participants were warned that they had responded too quickly (“too fast” in red text for responses under 100 ms) or too slowly (“miss” in red text for responses over 1,500 ms). In this phase, there were two randomly interleaved QUEST staircases (Watson & Pelli, 1983) with 40 trials each: one staircase for each motion direction. Each QUEST had a starting value of 70% coherence and was restricted to values between 1% and 100% coherence. A threshold estimate was obtained for each QUEST using the mode of the posterior distribution, and these estimates were averaged to provide a single threshold estimate.

This threshold estimate was then used as the stimulus level for the main experimental phase, which consisted of five blocks of 40 trials. In this phase, a central cue (width 0.8°) was presented for 1,000 ms before the motion stimulus, with a fixation cross presented for 500 ms before and after the cue (Figure 2b). Half of the trials had neutral, non-directional cues (a square), while the other half of the trials had directional arrow cues. 80% of the arrow cues validly predicted the upcoming motion direction, while the remaining 20% of the arrow cues were invalid. The stimuli moved leftward on half of the trials. Trial order was randomised within each block. Participants were advised that the arrows would indicate the most likely direction of the upcoming stimulus, but that the squares would not give any information about the upcoming stimulus. As before, participants were asked to respond as accurately and quickly as possible and trial-by-trial feedback was given. In addition, participants saw their total number of points at the end of each block. The experimental code can be found at https://osf.io/jqpxy/. Participants also completed an online or paper version of the AQ (Baron-Cohen et al., 2001) yielding a total score ranging from 0 to 50.

### Data filtering

Trials with very fast (under 200 ms), or very slow (over 1,500 ms), response times were removed from each participant’s data set. An average of 98.22% of trials were retained (range: 54.5%–100%).

### Pre-registered analyses (Model 1)

**Figure 3.**
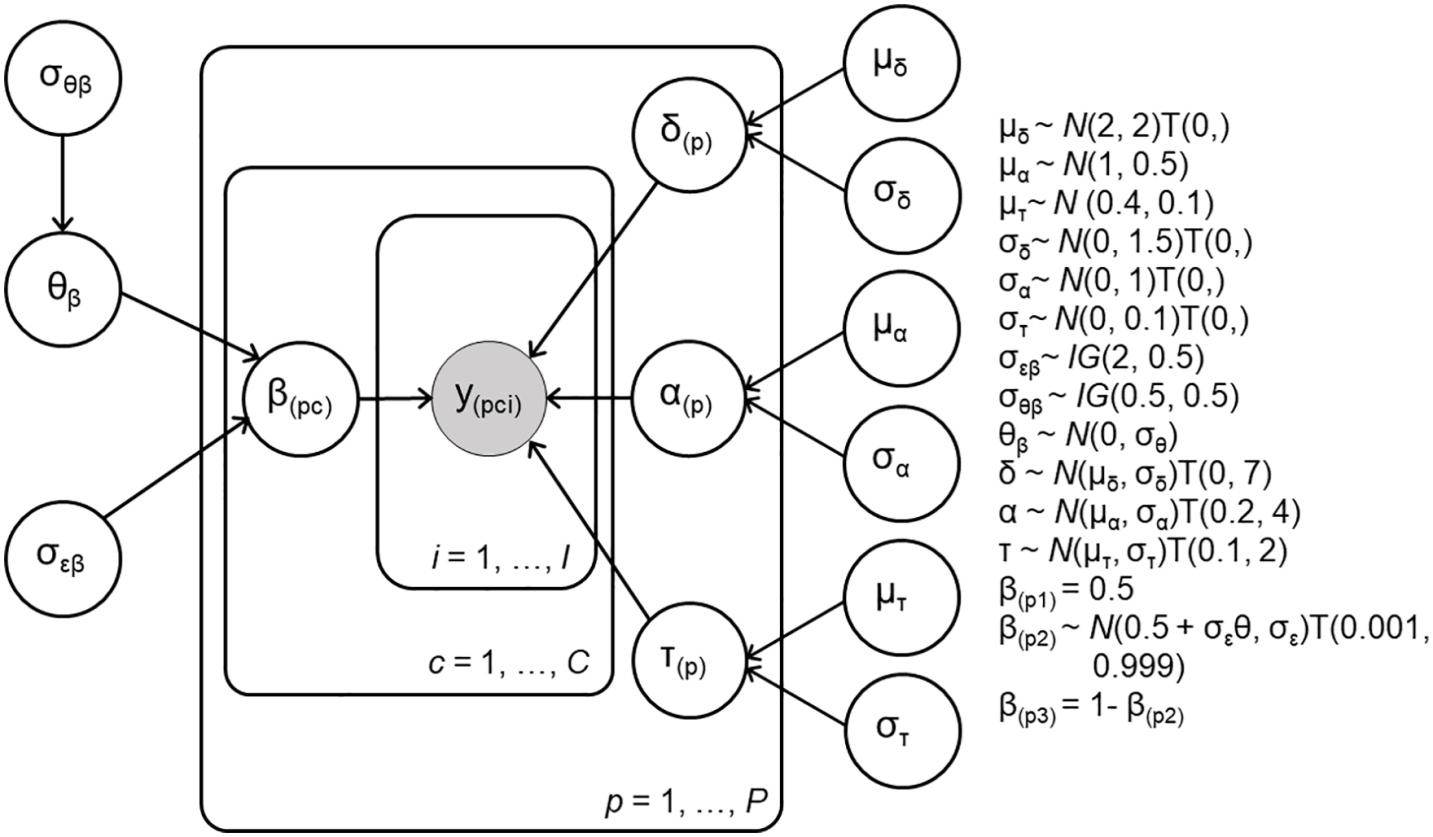
Graphical representation of Model 1 with condition effect on starting point. The data y_(pci)_ for each participant *p*, condition *c*, and trial *i* were assumed to be distributed according to the diffusion model’s first passage time distribution with drift rate δ, boundary separation α, non-decision time τ and starting point β parameters. In the neutral condition (*c* = 1), the starting point β is fixed at 0.5. Starting point β is estimated with a symmetric effect in valid (*c* = 2) and invalid (*c* = 3) conditions.

A Bayesian, hierarchical Wiener diffusion model (Vandekerckhove et al., 2011) was fit to the response time and accuracy data using Stan and the RStan package (Stan Development Team, 2020). Following Mulder et al. (2012), we used accuracy coding (collapsing across leftward and rightward stimuli), categorising trials as correct and incorrect trials in valid, invalid, and neutral conditions. As Mulder et al. (2012) reported that prior probability information biases the starting point (but not the drift rate), our pre-registered analyses modelled only a condition effect on starting point. The drift rate δ, boundary separation α, and non-decision time τ were estimated for each participant with population means μ__δ__, μ__α__, μ__τ__ and variances $\sigma_{\delta }^{2},\sigma_{\alpha }^{2},\sigma_{\tau }^{2}$ . The priors for the group-level distributions were based on previous work (Manning et al., 2021; Matzke & Wagenmakers, 2009) and participant-level prior distributions were truncated to ensure plausibility. Following Mulder et al. (2012), the starting point for the neutral cue trials (condition *c* = 1; β_*(p*1)_) was fixed at 0.5 (i.e., exactly halfway between the correct and incorrect boundary). The starting point for valid and invalid trials was modelled with a symmetric condition effect, such that valid cues bias the participant towards the correct response and invalid cues bias the participant towards the incorrect response with the same magnitude. The condition effect was modelled using Rouder et al.’s (2012) Bayesian analysis of variance (ANOVA) framework. For valid trials (condition *c* = 2), the starting point β_(*p*2)_ for participant *p* was drawn from a truncated normal distribution with a mean 0.5 + θσ__ε__, where θ is the standardised effect size and σ__ε__ is the residual standard deviation. For invalid trials (condition *c* = 3), the starting point was 1 − β_(*p*2)_. A graphical representation of Model 1 is presented in Figure 3.

We sampled from the posterior distribution using three parallel chains with 15,000 iterations, each with a burn in period of 6,000 samples, resulting in 27,000 retained samples. Starting values for all parameters were drawn from uniform distributions within the range of admissible parameter values. We posted the model on the Open Science Framework before investigating relationships with AQ, to ensure that our analyses were not biased according to our hypotheses. Finally, we used a plausible values approach (Ly et al., 2017) to investigate relationships between AQ scores and our parameters of interest (change in starting point across conditions β_(*p*2)_ − 0.5, and boundary separation α). Rather than performing frequentist correlational analyses on point estimates derived from the posterior distribution, the plausible values approach takes account of both uncertainty in the posterior distribution and uncertainty in generalising from a sample to the population. Briefly, correlations between each of 3,000 posterior draws for a given parameter and AQ were computed, resulting in a distribution of plausible correlations. For each plausible correlation, the implied analytic posterior population distribution was computed and subsequently averaged across plausible draws (Ly et al., 2018; https://github.com/AlexanderLyNL/bstats). We then computed the 95% equal tail credible interval for the posterior distribution of the population correlation and the Bayesian *p*-value, defined as the area of the posterior distribution above or below zero (Klauer, 2010; Skippen et al., 2019). A 95% credible interval encompassing zero suggests no reliable correlation. Models, data, and analysis scripts are available at https://osf.io/jqpxy/

### Exploratory analyses

Some areas of misfitting were identified in the posterior predictive plots for our pre-registered model (Model 1, Supplementary Figure S1), particularly at the shortest and longest RT quantiles, with the model predicting wider response time distributions than those present in the data. We therefore built two further models to see if these would provide a better fit to the data. Model 2 had a symmetric condition effect on drift rate δ while starting point β was fixed at 0.5 for all conditions (see Supplementary Figure S2). For each participant *p* in condition *c*, drift rate (δ_(*pc*)_) was drawn from a truncated normal distribution with a mean determined by μ_δ_ and a standardised effect size with standard deviation σ_ε._ Model 3 had condition effects on both starting point β and drift rate δ (see Supplementary Figure S3). We computed log marginal likelihoods for each model with warp-III bridge sampling (Gronau, Heathcote, & Matzke, 2020; Meng & Schilling, 2002; Meng & Wong, 1996), using the bridgesampling R package (Gronau, Singmann, & Wagenmakers, 2020), with six repetitions and a maximum of 20,000 iterations. Based on the marginal likelihoods, we then calculated Bayes factors to determine the most plausible model.

## Results

### AQ, accuracy, and response time

The coherence level presented to participants in the experimental phase based on their staircase trials ranged between 0.06 and 1 (*M* = 0.71, *SD* = 0.31). The coherence level presented did not correlate with AQ, *r*(220) = −0.02, 95% CI = [−0.15, 0.11], showing that the adaptive procedure did not lead to important between-participants differences as a function of AQ.

**Figure 4.**
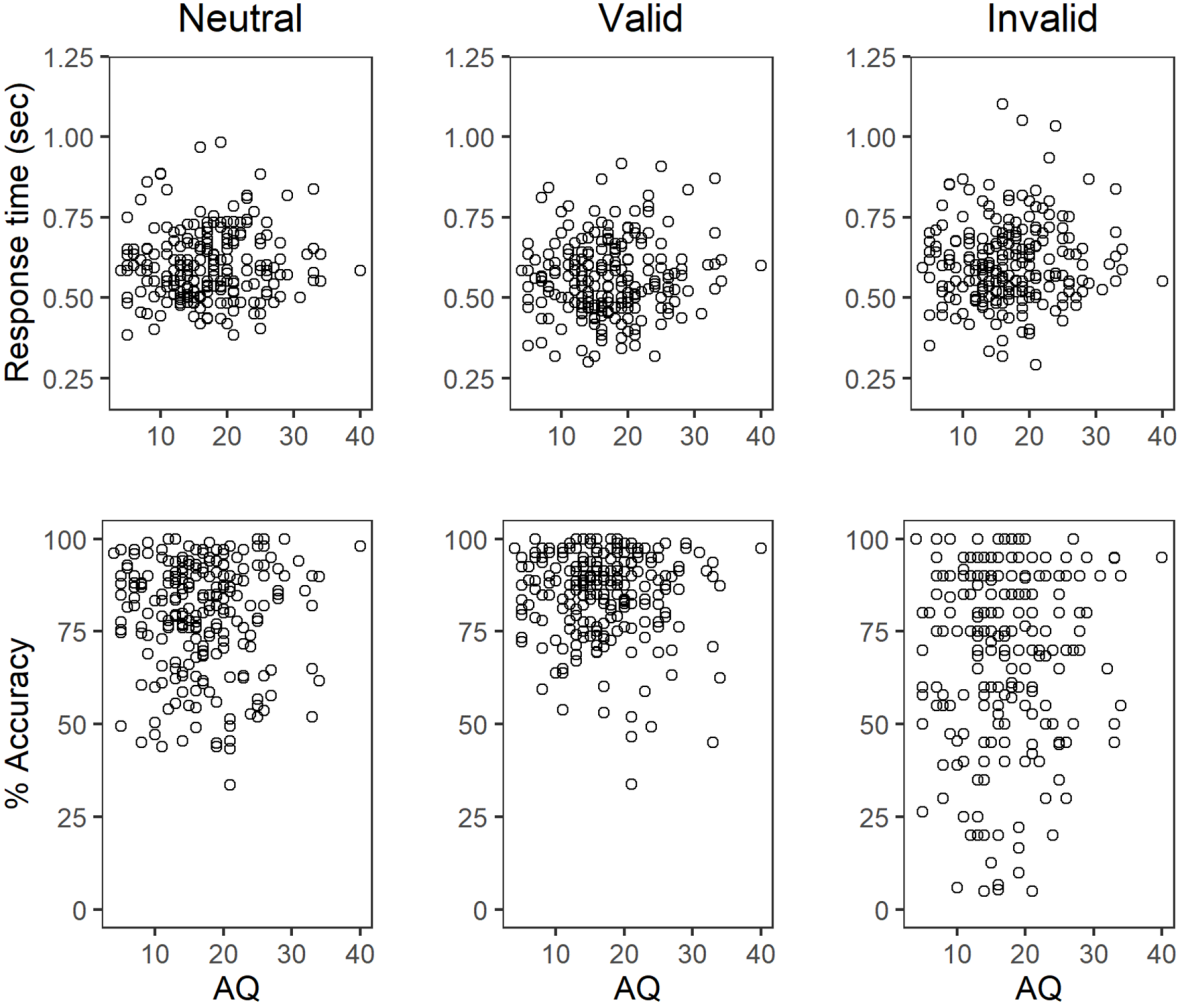
Scatterplots showing the relationship between autism spectrum quotient (AQ) scores and accuracy and median response times for correct trials.

Table 1 shows the mean of participants’ median RTs for correct trials and accuracy across cue conditions. As expected, accuracy was highest for valid trials and lowest for invalid trials, and response times were slightly faster in valid trials than in neutral and invalid trials. Figure 4 plots accuracy and median RT as a function of AQ score, and Table 2 presents correlation coefficients between these performance measures and AQ score. As shown, accuracy and RT did not appear to vary systematically with AQ score.

**Table 1.** Task performance metrics.

Measure	Cue type		
	Neutral	Valid	Invalid
Median RT (*SD*)	0.60 (0.11)	0.56 (0.12)	0.61 (0.13)
Accuracy (SD)	79.21 (15.02)	85.01 (11.78)	68.72 (23.57)

RT: response time; SD: standard deviation.

Mean and standard deviation of median response time (RT) for correct trials in seconds and percentage accuracy for each cue type.

**Table 2.** Correlations between autism spectrum quotient (AQ) scores and accuracy and median response times for correct trials following neutral, valid, and invalid cues.

Measure	Correlation with AQ	
	*R*	CI
Median RT		
Neutral	0.05	[−0.08, 0.18]
Valid	0.08	[−0.05, 0.21]
Invalid	0.06	[−0.07, 0.19]
% Accuracy		
Neutral	−0.02	[−0.15, 0.11]
Valid	−0.05	[−0.18, 0.09]
Invalid	0.05	[−0.09, 0.18]

AQ: Adult Autism Spectrum Quotient; CI: 95% confidence intervals; RT: response time.

### Pre-registered diffusion model analysis

**Figure 5.**
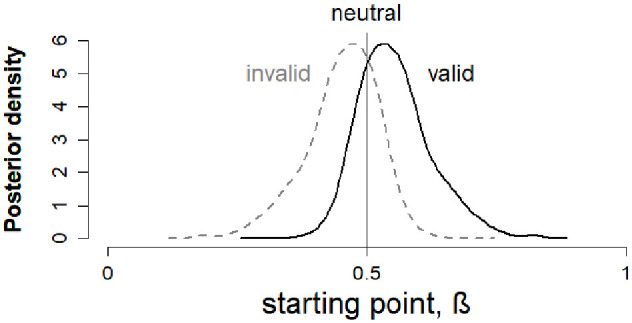
Posterior density of starting point values for valid and invalid cue conditions in Model 1. In the pre-registered analyses (Model 1), starting point β for the neutral condition was fixed at 0.5 and a symmetric effect in valid and invalid cue conditions was modelled, with the posterior distribution shifting towards 1 (the correct boundary) in the valid condition, and towards 0 (the incorrect boundary) in the invalid condition.

**Figure 6.**
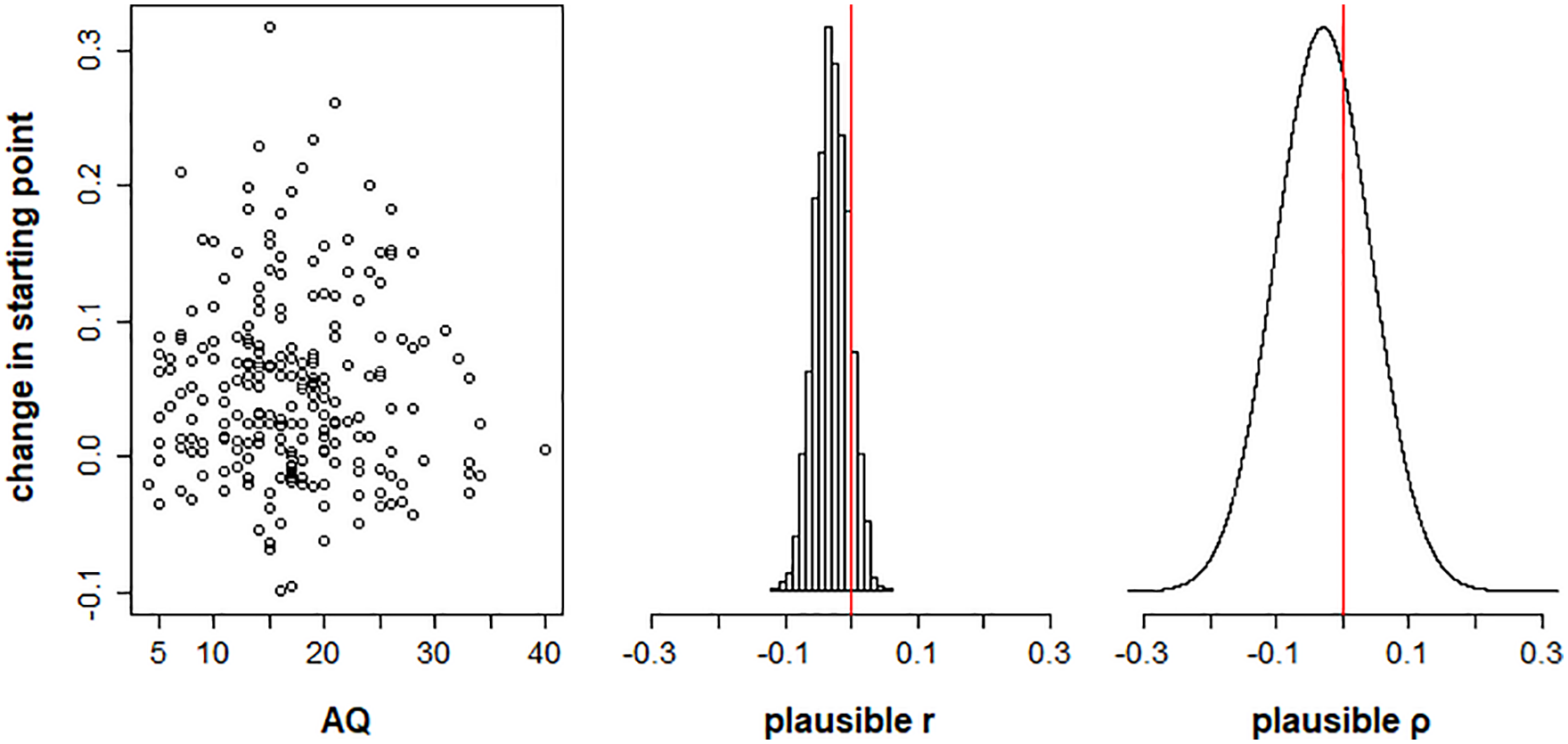
Plots showing the relationship between autism spectrum quotient (AQ) scores and change in starting point between validly cued and neutral trials, for Model 1. The left panel plots posterior mean estimates of change in starting point (β_(*p*2)_ − 0.5) for each participant as a function of AQ score. The middle panel shows the distribution of plausible correlations r between change in starting point and AQ in the sample. The right panel shows the posterior distribution of the plausible population correlation, ρ.

Our pre-registered analysis used a model (Model 1) with a condition effect on starting point (Figure 3). The model converged well, with Gelman–Rubin diagnostic values (Gelman & Rubin, 1992) close to 1 (range = 0.999–1.000). Posterior predictives for this model can be found in Supplementary Figure S1. Overall, starting point varied with condition in the expected direction, with valid cues biasing responses towards the correct response and invalid cues biasing responses towards the incorrect response (see Figure 5). However, as shown in Figure 6 (left panel), some participants showed a change in starting point in the opposite direction, which may reflect measurement error due to the relatively small number of trials for each participant.

Our first hypothesis was that the change in starting point between neutral and cued conditions (i.e., β_(*p*2)_ – 0.5) would vary as a function of autistic traits. Figure 6 shows the plausible correlations and population correlation distribution for this relationship. The 95% equal tail credible interval for the population correlation posterior distribution spanned zero [−.17, .11] (Bayesian *p*-value = .34), suggesting no reliable relationship between AQ and effect of prior information on starting point.

**Figure 7.**
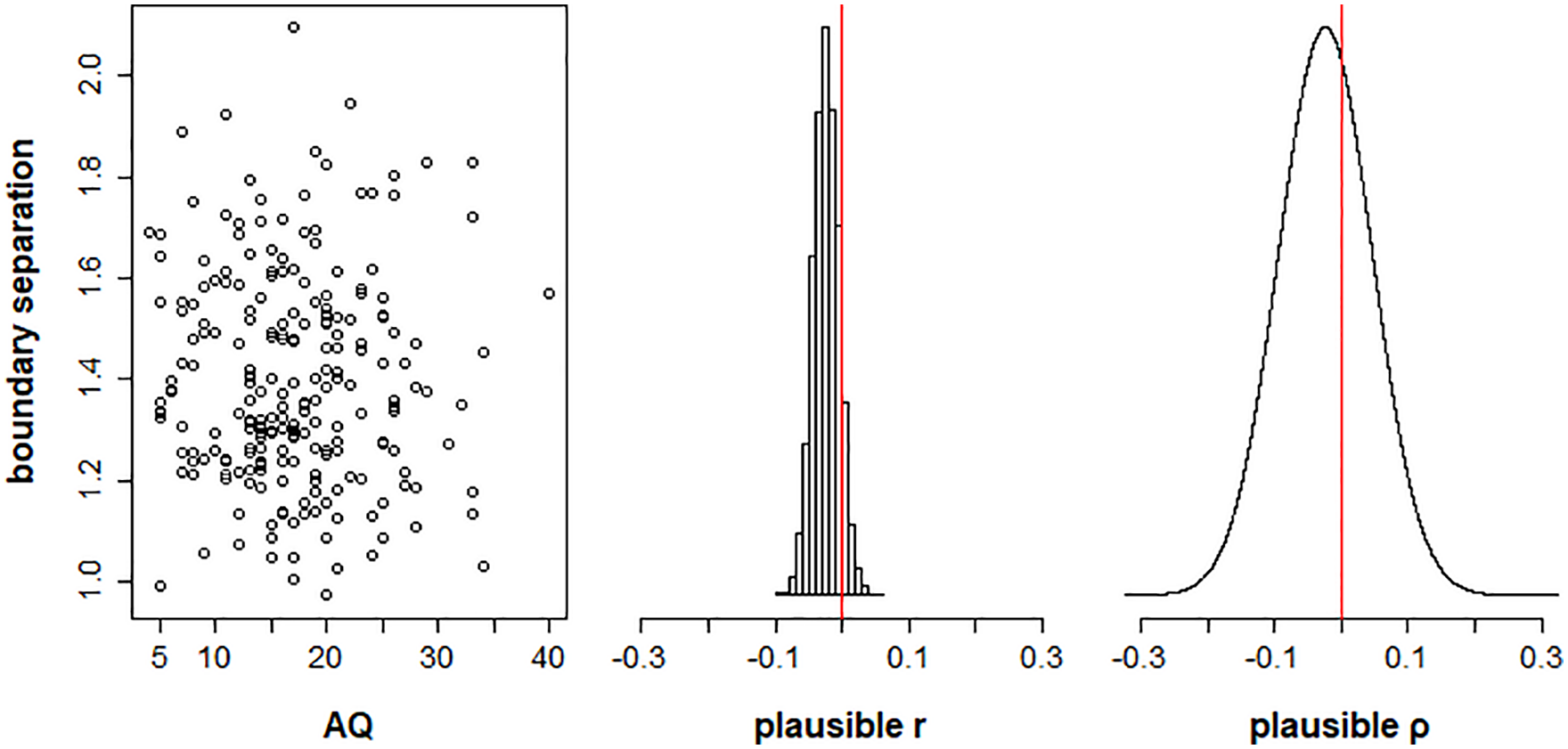
Plots showing the relationship between autism spectrum quotient (AQ) scores and boundary separation between validly cued and neutral trials, for Model 1. The left panel plots posterior mean estimates of boundary separation α for each participant as a function of AQ score. The middle panel shows the distribution of plausible correlations r between boundary separation and AQ in the sample. The right panel shows the posterior distribution of the plausible population correlation, ρ.

Our second hypothesis was that boundary separation α would correlate with AQ scores. Again, we found no evidence for this relationship (Figure 7), with the population correlation posterior distribution having a 95% equal-tailed credible interval of [−.16, .11] (Bayesian *p*-value = .37).

### Exploratory analyses

As the posterior predictives for Model 1 showed some areas of mis-fitting, we compared this model with Model 2, which allowed drift rate to vary across cue conditions while starting point was fixed at 0.5 (Supplementary Figure S2), and Model 3, which allowed both drift rate and starting point to vary according to cue conditions (Supplementary Figure S3). Posterior predictives for these additional models can be found in Supplementary Figures S4 and S5. It is worth noting that the same pattern of misfitting found with Model 1 in the most extreme RT quantiles is also evident for these models. Bridge sampling estimated the log marginal likelihood of Models 1 to 3 to be −5,076.57, −4,956.39, and −4,863.51, respectively. Both Model 2 (with a condition effect on drift rate) and Model 3 (with a condition effect on both drift rate and starting point) were preferred over Model 1 (which had a condition effect only on starting point), with log Bayes factors of 120.19 for the comparison between Models 2 and 1, and 213.07 for the comparison between Models 3 and 1. Out of the two additional models, Model 3 was preferred (log Bayes factor = 92.89), suggesting that a model with condition effects on both drift rate and starting point provided the best fit to the data. The posterior mean for drift rate was slightly higher in the valid cue condition (*M* = 1.59, *SD* = 0.79) than in the neutral condition (*M* = 1.33, *SD* = 0.83), showing that sensory evidence accumulation was accelerated following valid cues. Importantly, when applying the plausible values approach to the posterior samples from this model, we still did not find evidence to support our hypotheses (see Supplementary Figures S6 and S7). We also investigated whether change in drift rate related to AQ. There was no clear evidence of a relationship (see Supplementary Figure S8) with a 95% equal-tailed credible interval of [−.20, .08] (Bayesian *p*-value = .19).

## Discussion

In this study, we investigated how perceptual decision-making varies as a function of autistic-like traits in a large sample of the general population using a diffusion model framework. We used a motion direction discrimination task with probabilistic cues that have been reported to bias the starting point of the decision-making process, meaning that less sensory evidence is required to trigger a response towards the cued direction (Mulder et al., 2012). Following accounts of reduced reliance on prior information in autism (Pellicano & Burr, 2012), we hypothesised that starting point biases would be reduced in people with high levels of autistic-like traits compared with those with low levels of autistic-like traits. Yet, we found no evidence for a relationship between starting point bias and autistic-like traits. Similarly, we found no evidence for our hypothesis that individuals with high levels of autistic-like traits would make more cautious responses (with wider decision boundaries) than individuals with low levels of autistic-like traits. We consider three possible interpretations of these results.

First, our results may suggest that the proposed differences in prior information use and response caution in autism (Pellicano & Burr, 2012; Pirrone et al., 2017; Pirrone et al., 2020) do not extend to individual differences in autistic-like traits in the general population. Although some previous studies have found support for the weakened priors account in non-clinical populations with high levels of autistic-like traits (Powell et al., 2016; Skewes et al., 2015), others have not (Andermane et al., 2020; Ewbank et al., 2016; Tulver et al., 2019). Pirrone et al. (2018) reported previously that increased response caution did not vary as a function of autistic-like traits in the general population, which we also report here in a larger sample with a different paradigm. It is possible that our staircase procedure designed to titrate difficulty levels across participants may have inadvertently influenced boundary separation settings across the sample, so extensions of this paradigm could be optimised for assessing boundary separation differences as a function of AQ. Previous failures to extend findings from autistic populations to non-autistic populations have challenged the idea that the perceptual differences found in autism vary along a continuum across the whole population (Gregory & Plaisted-Grant, 2016). Perhaps, more generally, autism should be conceptualised as a qualitatively distinct category relative to the typical population (e.g., Frazier et al., 2010). It is therefore crucial to present our task and modelling approach to diagnosed autistic individuals to contextualise the clinical relevance of our result.

Second, there are methodological limitations with the AQ that may obscure relationships with perceptual decision-making parameters. First, AQ scores do not correspond perfectly to diagnoses, with many autistic people scoring below the cut-off (Ashwood et al., 2016; Bishop & Seltzer, 2012), and no clear relationship between AQ scores and standard clinical measures (Bishop & Seltzer, 2012). It has also been suggested that the construct measured by the AQ is not continuous, as assumed, but instead categorical (James et al., 2016), making it difficult to interpret relationships (or lack thereof) with the total AQ score (see also English et al., 2020). Similarly, Agelink van Rentergem et al. (2019) reported that some of the items on the AQ function differently between autistic and non-autistic samples. Although alternative scoring algorithms have been proposed to mitigate some of these challenges (Agelink van Rentergem et al., 2019; English et al., 2020), we were unable to use these as we did not have access to item-level data from all participants. Yet, despite the conceptual and methodological issues associated with the AQ, some studies have nonetheless reported relationships between AQ scores and prior information use in different tasks (Powell et al., 2016; Skewes et al., 2015).

This discrepancy leads us to our final interpretation of our results, which is that only some types of prior information use are altered in autism. Accordingly, mixed findings are emerging from studies comparing diagnosed autistic and non-autistic individuals (e.g., Croydon et al., 2017; Karaminis et al., 2016; Pell et al., 2016; Skewes & Gebauer, 2016; Van de Cruys et al., 2018). This mixed pattern of results could reflect the fact that there are many different types of prior information which influence perception through different timescales (see Series & Seitz, 2013, for review), which may be selectively affected in autism. For example, in our study, the prior information is explicit and immediately precedes the stimulus, changing from trial-to-trial, whereas in other studies the prior distribution is implicitly learned over a block of trials (e.g., Karvelis et al., 2018) or built up during experience of the world outside the experimental session (e.g., Powell et al., 2016). Interestingly, Tulver et al. (2019) found no single factor explaining individual differences in the effects of prior information in different perceptual tasks, arguing that priors can only be understood in reference to specific tasks and stimuli. Ultimately, large studies presenting multiple tasks with different types of prior information to both autistic and non-autistic individuals would inform us about which aspects of prior information use are atypical in autism, and which of these extend to individual differences in autistic-like traits. Such an investigation would in turn inform theoretical accounts regarding prior information use in autism. Moreover, future research will be required to investigate the level of processing at which autism-related differences arise: the extent to which prior information is processed, the extent to which it is incorporated into a mental representation, and/or the extent to which subsequent decision-making uses that prior information.

The results of our exploratory analyses also have implications for models of bias in perceptual decision-making tasks. Previous studies have reported that prior information about the likely upcoming motion direction (Forstmann et al., 2010; Mulder et al., 2012) or pain intensity (Wiech et al., 2014) biases the starting point of the decision-making process, rather than biasing the drift rate. According to these studies, prior information alters the decision-making process while leaving sensory processing unaffected—a suggestion which has been supported by single cell recordings in monkeys (Rao et al., 2012). Yet in our exploratory analyses, we found that a model allowing drift rate to vary following probabilistic cues was more plausible than a model with a change in starting point, with the most plausible model being one with effects on both drift rate and starting point. Although we are not the first to report bias in drift rate as a result of prior information (Hanks et al., 2011; van Ravenzwaaij et al., 2012), previous reports have used tasks where the prior probability of stimulus motion was manipulated within a block. The differences between our results and those obtained using a similar paradigm by Mulder et al. (2012) could be because Mulder et al. used a more complex model that can account for random trial-by-trial variability in bias. The importance of trial-by-trial fluctuations in bias could be modelled in future work using reinforcement learning models incorporating diffusion processes (Pedersen et al., 2017) or modified diffusion models where the starting point depends on the previous decision (Olianezhad et al., 2019). Importantly, attending to a certain direction can modulate the response of direction-selective cells (see Carrasco, 2011; Maunsell & Treue, 2006, for reviews) and change the gain and tuning of population responses (Ling et al., 2009), providing a potential mechanism for the effect of directional cues on drift rate that we report. However, as this result was not hypothesised, future confirmatory studies are required. Crucially, the model of bias chosen did not affect our conclusions: neither bias in starting point nor bias in drift rate related to autistic-like traits.

## Conclusion

In this study, we used a diffusion model framework to investigate whether perceptual decision-making characteristics vary as a function of autistic-like traits in the general population. We found no evidence for our hypotheses that individuals with high levels of autistic-like traits would incorporate prior information into their decisions to a lesser extent, and respond more cautiously, than those with low levels of autistic-like traits. With future application to diagnosed autistic individuals, this paradigm will be useful for probing the limits to theories proposing reduced prior information use in autism.

## Supplemental Material

sj-pdf-1-qjp-10.1177_17470218211019939 – Supplemental material for Prior information use and response caution in perceptual decision-making: No evidence for a relationship with autistic-like traits Supplemental material, sj-pdf-1-qjp-10.1177_17470218211019939 for Prior information use and response caution in perceptual decision-making: No evidence for a relationship with autistic-like traits by Chris Retzler, Udo Boehm, Jing Cai, Aimee Cochrane and Catherine Manning in Quarterly Journal of Experimental Psychology
