# Supplementary material for: Prior information use and response caution in perceptual decision-making: No evidence for a relationship with autistic-like traits
Source: Q J Exp Psychol (Hove). 2021 May 25;74(11):1953–65. doi: 10.1177/17470218211019939 (PMC8450985; doi:10.1177/17470218211019939)
Supplement: sj-pdf-1-qjp-10.1177_17470218211019939 – Supplemental material for Prior information use and response caution in perceptual decision-making: No evidence for a relationship with autistic-like traits [file sj-pdf-1-qjp-10.1177_17470218211019939.pdf]

**Supplementary Materials**

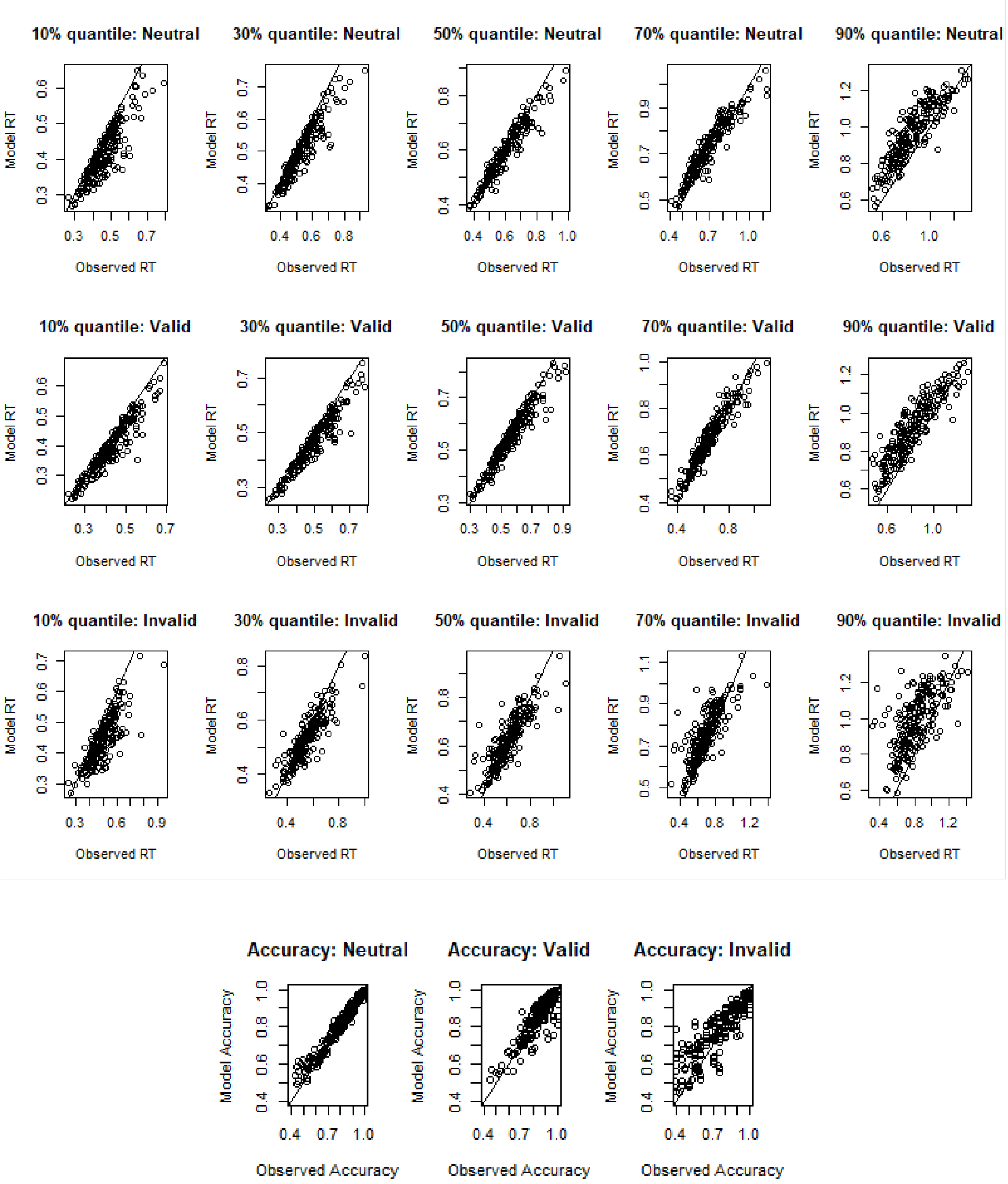


**Figure S1. Posterior predictive plots for Model 1 (pre-registered analysis) with a condition effect on starting point**

Observed response times for each quantile (10%, 30%, 50%, 70%, 90%) for correct trials and observed accuracy for each participant plotted against those predicted by the model, for each cue condition (neutral, valid, invalid). The diagonal line is the line y = x.

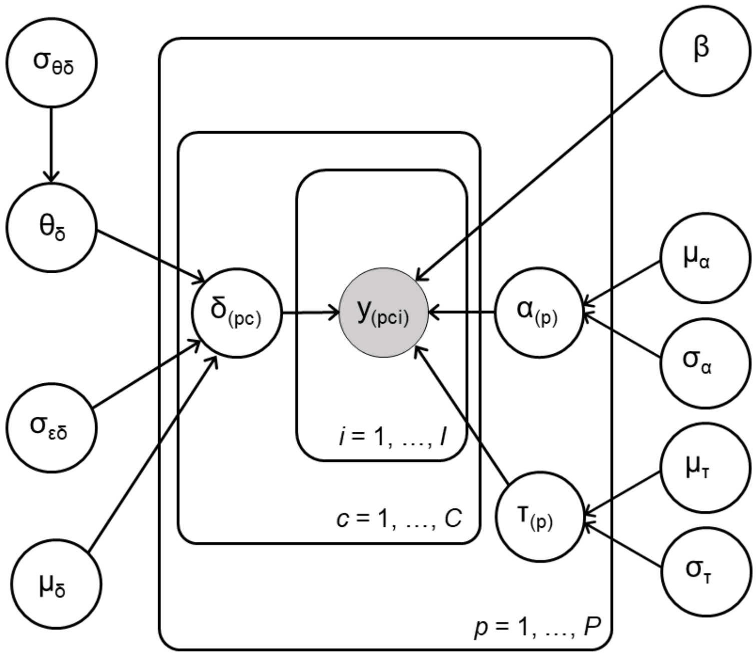


$\beta = 0.5$
$\mu_\alpha \sim N(1, 0.5)$
$\mu_\tau \sim N\ (0.4, 0.1)$
$\mu_\delta \sim N(2, 2)\mathrm{T}(0,)$
$\sigma_\alpha \sim N(0, 1)\mathrm{T}(0,)$
$\sigma_\tau \sim N(0, 0.1)\mathrm{T}(0,)$
$\sigma_{\varepsilon\delta} \sim IG(2, 0.5)$
$\sigma_{\theta\delta} \sim IG(0.5, 0.5)$
$\theta_\delta \sim N(0, \sigma_\theta)$
$\alpha \sim N(\mu_\alpha, \sigma_\alpha)\mathrm{T}(0.2, 4)$
$\tau \sim N(\mu_\tau, \sigma_\tau)\mathrm{T}(0.1, 2)$
$\delta_{(p1)} \sim N(\mu_\delta - \sigma_\varepsilon(2^{-1/2}\,\theta), \sigma_\varepsilon)\mathrm{T}(0, 7)$
$\delta_{(p2)} \sim N(\mu_\delta + \sigma_\varepsilon(2^{-1/2}\,\theta), \sigma_\varepsilon)\mathrm{T}(0, 7)$
$\delta_{(p3)} = 2\delta_{(p1)} - \delta_{(p2)}$

**Figure S2. Graphical representation of Model 2 presented in exploratory results with a condition effect on drift rate**

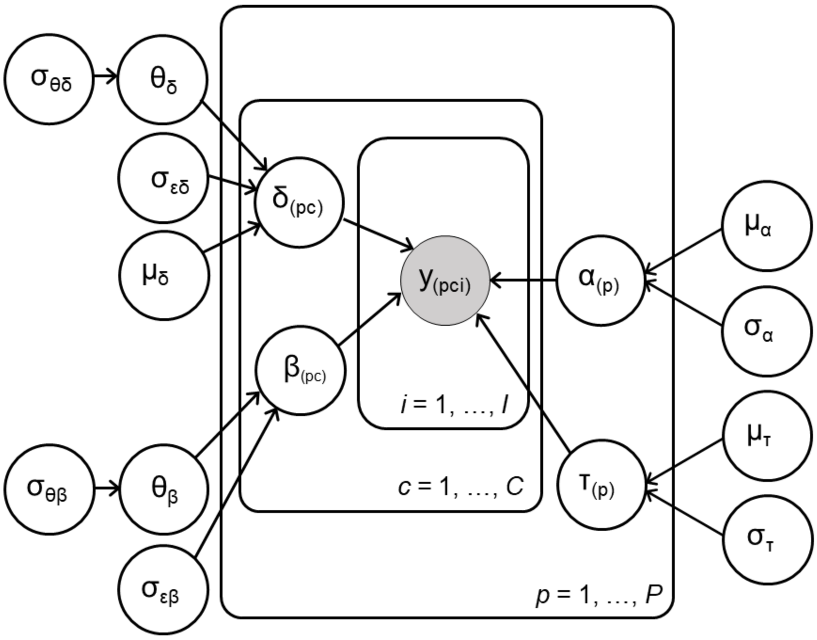


$\mu_\alpha \sim N(1, 0.5)$
$\mu_\tau \sim N\ (0.4, 0.1)$
$\mu_\delta \sim N(2, 2)\mathrm{T}(0,)$
$\sigma_\alpha \sim N(0, 1)\mathrm{T}(0,)$
$\sigma_\tau \sim N(0, 0.1)\mathrm{T}(0,)$
$\sigma_{\varepsilon\delta} \sim IG(2, 0.5)$
$\sigma_{\varepsilon\beta} \sim IG(2, 0.5)$
$\sigma_{\theta\delta} \sim IG(0.5, 0.5)$
$\sigma_{\theta\beta} \sim IG(0.5, 0.5)$
$\theta_\delta \sim N(0, \sigma_{\theta\delta})$
$\theta_\beta \sim N(0, \sigma_{\theta\beta})$
$\alpha \sim N(\mu_\alpha, \sigma_\alpha)\mathrm{T}(0.2, 4)$
$\tau \sim N(\mu_\tau, \sigma_\tau)\mathrm{T}(0.1, 2)$
$\delta_{(p1)} \sim N(\mu_\delta - \sigma_{\varepsilon\delta}(2^{-1/2}\,\theta_\delta), \sigma_{\varepsilon\delta})\mathrm{T}(0, 7)$
$\delta_{(p2)} \sim N(\mu_\delta + \sigma_{\varepsilon\delta}(2^{-1/2}\,\theta_\delta), \sigma_{\varepsilon\delta})\mathrm{T}(0, 7)$
$\delta_{(p3)} = 2\delta_{(p1)} - \delta_{(p2)}$
$\beta_{(p1)} = 0.5$
$\beta_{(p2)} \sim N(0.5 + \sigma_\varepsilon\theta, \sigma_\varepsilon)\mathrm{T}(0.001, 0.999)$
$\beta_{(p3)} = 1 - \beta_{(p2)}$

**Figure S3. Graphical representation of Model 3 presented in exploratory results with condition effects on both starting point and drift rate**

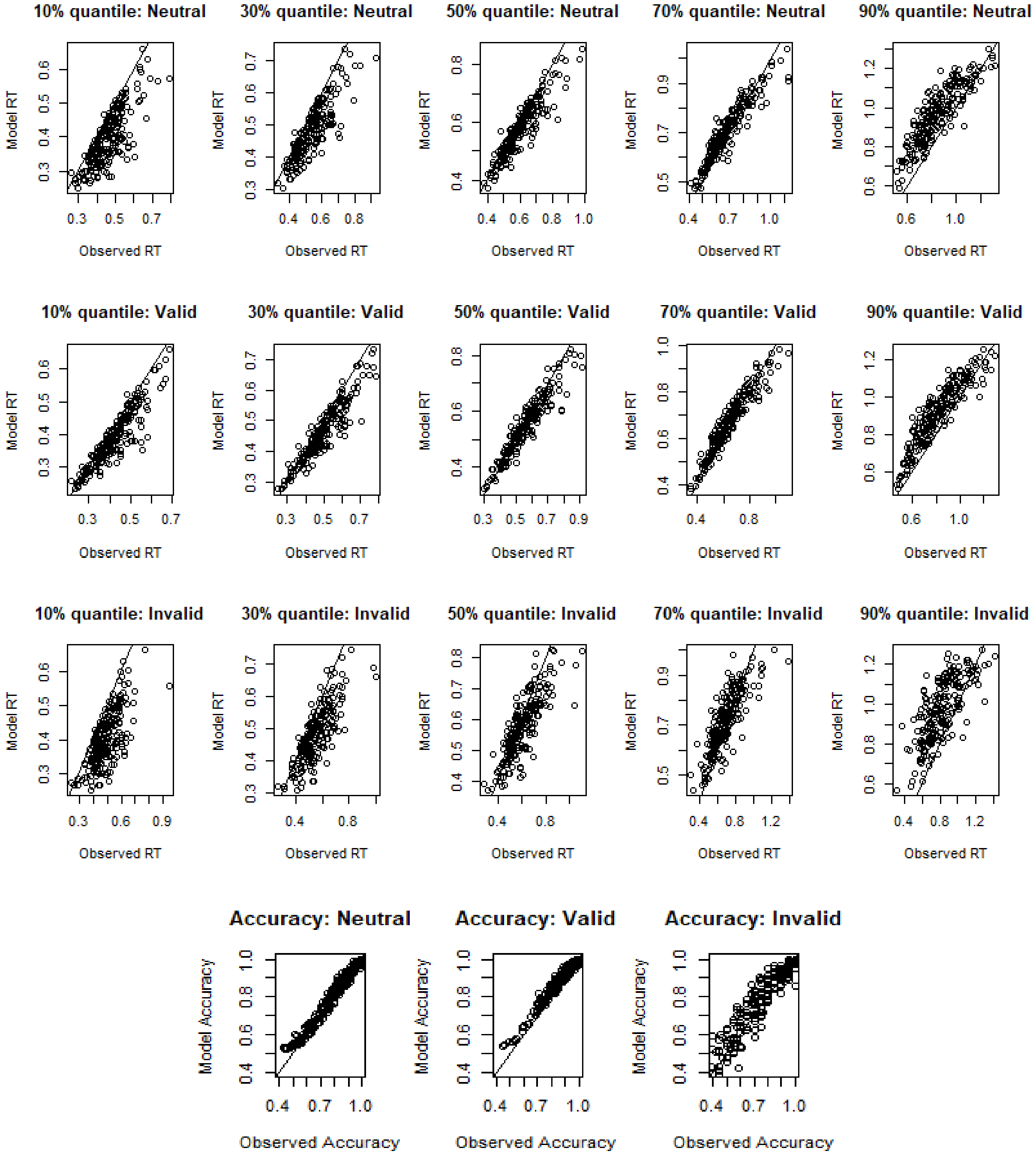


**Figure S4. Posterior predictives for Model 2 with a condition effect on drift rate**

Observed response times for each quantile (10%, 30%, 50%, 70%, 90%) for correct trials and observed accuracy for each participant plotted against those predicted by the model, for each cue condition (neutral, valid, invalid). The diagonal line is the line y = x.

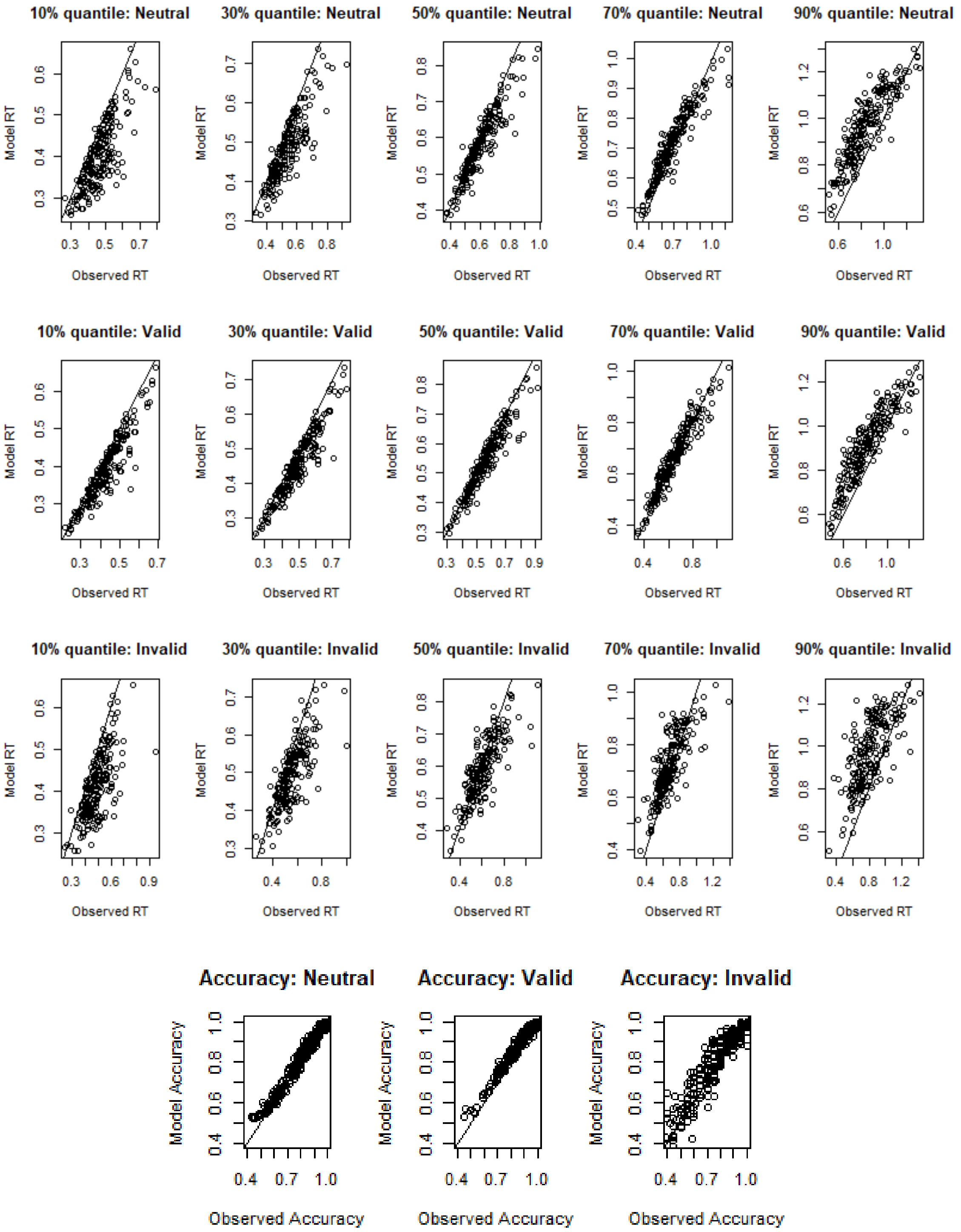


**Figure S5. Posterior predictives for Model 3 with a condition effect on both starting point and drift rate**

Observed response times for each quantile (10%, 30%, 50%, 70%, 90%) for correct trials and observed accuracy for each participant plotted against those predicted by the model, for each cue condition (neutral, valid, invalid). The diagonal line is the line y = x.

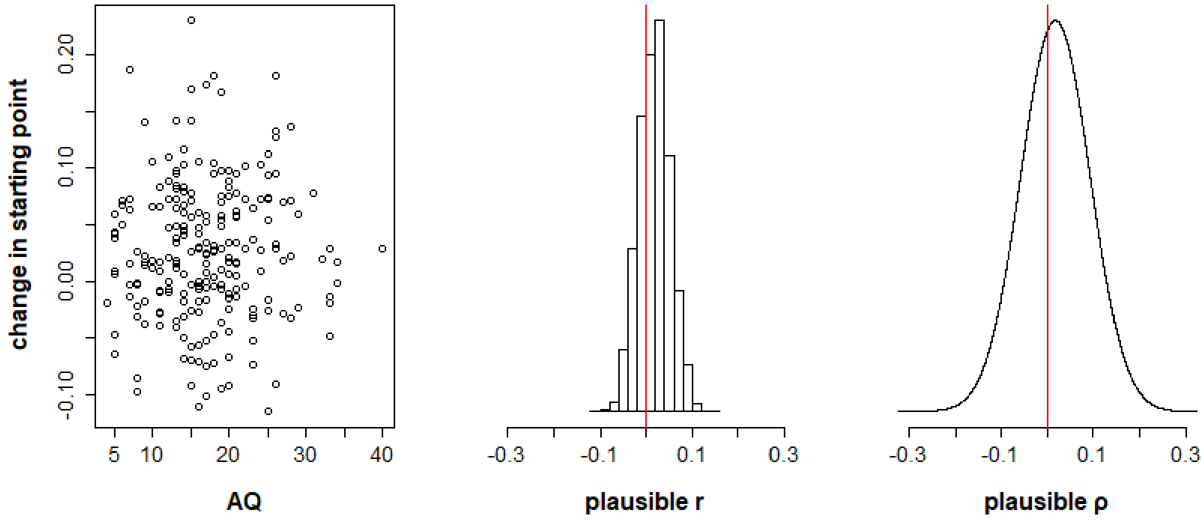


**Figure S6. Plots showing the relationship between autism spectrum quotient (AQ) scores and change in starting point between validly cued and neutral trials, for Model 3.**

The left panel plots posterior mean estimates of change in starting point (i.e., $\beta_{(p2)} - 0.5$) for each participant as a function of AQ score. The middle panel shows the distribution of plausible correlations *r* between change in starting point and AQ in the sample. The right panel shows the distribution of the plausible population correlation, ρ. The 95% equal tail credible interval spanned 0 ([-0.13, 0.16], Bayesian p-value = 0.41).

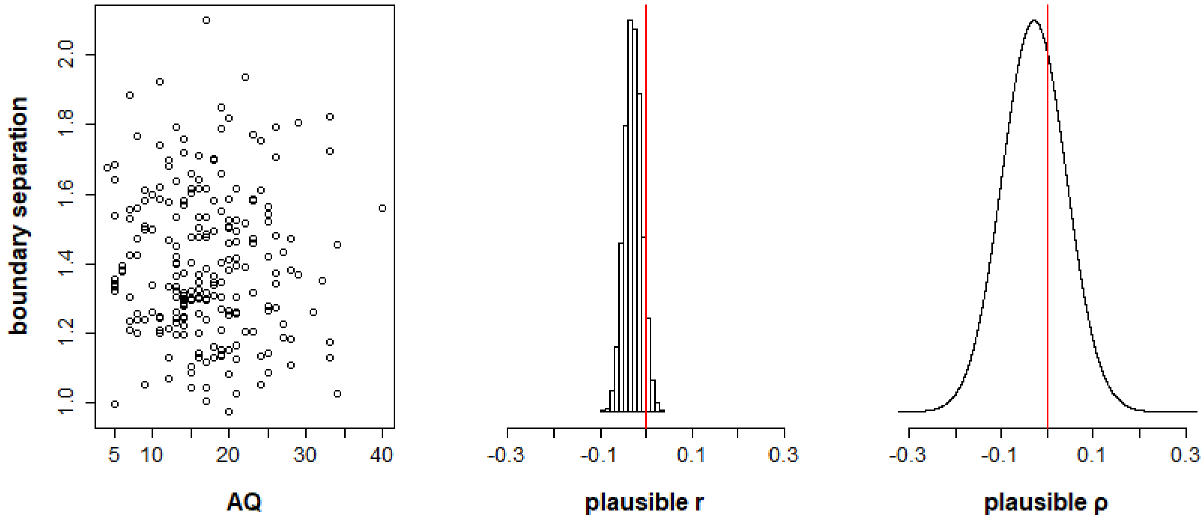

**Figure S7. Plots showing the relationship between autism spectrum quotient (AQ) scores and boundary separation, for Model 3.**

The left panel plots posterior mean estimates of boundary separation α for each participant as a function of AQ score. The middle panel shows the distribution of plausible correlations *r* between boundary separation and AQ in the sample. The right panel shows the distribution of the plausible population correlation, ρ. The 95% equal tail credible interval spanned 0 ([-0.16, 0.11], Bayesian p-value = 0.34).

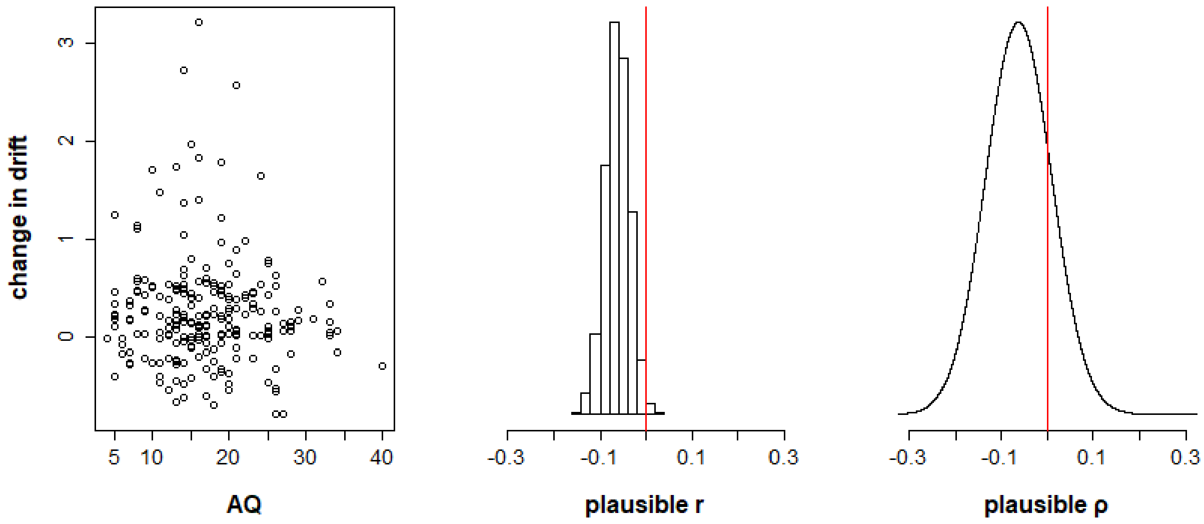


**Figure S8. Plots showing the relationship between autism spectrum quotient (AQ) scores and change in drift rate, for Model 3.**

The left panel plots posterior mean estimates of change in drift rate ($\delta_{(p2)} - \delta_{(p1)}$) for each participant as a function of AQ score. The middle panel shows the distribution of plausible correlations *r* between change in drift rate and AQ in the sample. The right panel shows the distribution of the plausible population correlation, ρ.
